# Supplementary figures and images for: Oncological Benefit versus Cardiovascular Risk in Breast Cancer Patients Treated with Modern Radiotherapy
Source: J Clin Med. 2022 Jul 4;11(13):3889. doi: 10.3390/jcm11133889 (PMC9267636; doi:10.3390/jcm11133889)

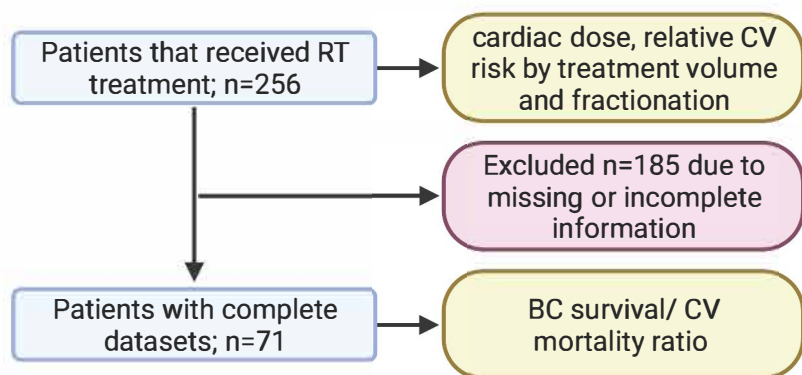

Figure S1: Patient flow chart according to inclusion/exclusion criteria.

Supplement: Supplementary file 1 [file jcm-11-03889-s001.zip › jcm-1763566-supplementary.pdf]
